# Overlap of Characteristic Serological Antibodies in Rheumatoid Arthritis and Wheat-Related Disorders

**DOI:** 10.1155/2019/4089178

**Published:** 2019-01-10

**Authors:** Yuanyuan Yang, Payal Deshpande, Karthik Krishna, Vinodh Ranganathan, Vasanth Jayaraman, Tianhao Wang, Kang Bei, John J. Rajasekaran, Hari Krishnamurthy

**Affiliations:** ^1^Vibrant America LLC., San Carlos, CA, USA; ^2^Vibrant Sciences, LLC., San Carlos, CA, USA

## Abstract

**Background and Aims:**

Rheumatoid arthritis (RA) and celiac disease (CD) are members of the autoimmune disease family while they have been shown to share multiple aspects in epidemiology and clinical manifestations. The aim of this study was to assess the presence of wheat protein antibodies in RA seropositive subjects and the presence of RA diagnostic markers in subjects with seropositive wheat-related disorders including CD.

**Methods:**

Serum samples were collected from 844 subjects with joint pain and/or gastrointestinal symptoms and tested by a CD panel (anti-tTG and anti-DGP), a Wheat Zoomer (WZ) antibody panel (IgG/IgA to 14 wheat proteins), and a RA panel (anti-CCP and anti-RF). Retrospective analysis was completed using de-identified clinical data and test results.

**Results:**

The prevalence of RA markers was first investigated in CD- or WZ-positive subjects and negative controls. 49 subjects were seropositive in the CD panel with 10 (20%) RA positivity. 605 subjects were seropositive in the WZ panel with 106 (18%) RA positivity. 222 subjects were seronegative in either panels with 12 (6%) RA positivity. Next, the frequency of the CD markers and the clinically relevant wheat protein antibodies were investigated in the RA-positive subjects and negative controls. 128 subjects in this cohort were seropositive in the RA panel with 10 (8%) CD positivity and 106 (83%) WZ positivity, compared to 716 RA seronegative controls with 39 (5%) CD positivity and 499 (70%) WZ positivity.

**Conclusions:**

Our data presents an apparent trend of overlapped serological antibody biomarker positivity in RA and wheat-related disorders.

## 1. Introduction

Rheumatoid arthritis (RA) is a systemic autoimmune disease that causes progressive articular damage, functional loss, and comorbidity. RA manifests as pain, stiffness, swelling, and functional impairment in the joints and is characterized by persistent synovitis, chronic inflammatory, and autoantibodies. As symptoms of RA can be closely mirroring other diseases, it is usually difficult to diagnose in the early stages. One of the most important and helpful criteria in reaching a RA diagnosis is to identify antibodies to rheumatoid factor (anti-RF) IgM and anti-cyclic citrullinated peptide (anti-CCP) IgG/IgA, which are known triggers of RA symptoms. In an established case, anti-RF IgM can be detected with a sensitivity of 60–70% and a specificity of 80–90% while anti-CCP IgG/IgA can be detected at a much higher specificity (98%) and a similar sensitivity (68–80%) [1]. Despite unknown etiology, new interest has emerged in studying the interaction of RA with other diseases due to the new opportunity for development of novel diagnostic and therapeutic strategies for the patients.

Wheat-related disorders occur in a broad spectrum of conditions by the ingestion of wheat and its major storage protein, gluten. Individuals usually develop gastrointestinal symptoms such as abdominal pain, diarrhea, and bloating. Celiac disease (CD), as the most common wheat-related disorder, is an autoimmune disorder precipitated in genetically predisposed individuals, and it affects about 1% of adults and children in the United States [2]. Adherence to a gluten-free diet has been so far the only proven treatment for CD [3]. Currently, the diagnosis of CD is relied on the presence of anti-tissue transglutaminase (tTG) IgA and deaminated gliadin peptide (DGP) IgA followed by histologic examination. Other than CD, we are currently witnessing a significant proportion of individuals encountering gastrointestinal and extraintestinal symptoms upon wheat ingestion in the absence of CD and wheat allergy. Numerous studies have been implemented to understand the pathogenesis of the nonceliac wheat sensitivity although there remain no sensitive and specific biomarkers [4].

Despite being separate entities, RA and CD share multiple aspects in epidemiology and clinical manifestations. The epidemiology of both disorders has been proven to be influenced by comparable environmental factors and recent incidental surge of associated antibodies. The infectious, dysbiosis, and increased intestinal permeability theories, as drivers of autoimmune cascade, apply to both CD and RA. They share some of the clinical manifestations such as CD extraintestinal rheumatic manifestations and RA gastrointestinal ones. There is also evidence that gluten-free diet may ameliorate celiac rheumatic manifestations but the theory of dietary effects on RA has not been determined [5]. Although RA and CD differ in HLA predispositions and specific diagnostic biomarkers, the pathophysiology of both diseases are both mediated by endogenous enzymes targeting different organs.

Though there have been numerous studies on similarities in the CD-RA interrelationship, the study on prevalence of CD or RA in each other's population have been underinvestigated and heterogeneous results have been reported. Lubrano et al. demonstrated a 26% prevalence of arthritis among 200 adult CD patients [5]. Francis et al. studied 160 British RA patients and found a single case of CD among them [6]. Neuhausen et al. observed that the number of CD cases in RA patients to be 1%, which is very similar to the number reported in the general population [7]. In another study of determining the frequency of celiac markers in 85 RA patients, anti-gliadin IgG antibodies were positive in 16 patients, anti-gliadin IgA in 29 patients, ultra-purified anti-gliadin in 14 patients, and only one patient had anti-tTG [8]. There are extensive studies evaluating the presence of CD characteristic biomarker in RA patients while studies on anti-CCP in CD patients are rare.

In this study, we investigated the overlap of characteristic biomarkers in RA and wheat-related disorders including CD. The aim of our study was to assess the presence of wheat protein antibodies to gluten and nongluten proteins in RA subjects and the presence of RA markers in subjects with wheat-related disorders. Understanding the biomarker overlap not only provides significant diagnostic importance but may ultimately contribute to understanding the pathophysiology of both diseases.

## 2. Materials and Methods

### 2.1. Study Design and Population

A total of 844 subjects with joint pain and/or gastrointestinal symptoms were tested in the Vibrant America Clinical Laboratory for the Celiac Disease panel, the Wheat Zoomer assay, and the Rheumatoid Arthritis panel between November 2015 and January 2018. No restricted diet was instructed to the subjects during the study period. None of the subjects has seropositive wheat allergy (wheat IgE positive). Mean age (±standard deviation) of the subjects was 47 ± 16 years. The female-to-male ratio was 2.4 : 1 (female 593, male 251).

To streamline the study, we defined the subjects into the following groups:

- Rheumatoid arthritis panel-positive (RA+) subjects—the subjects who were seropositive for at least one marker in the RA panel
- Rheumatoid arthritis panel-negative (RA−) controls—the subjects who were seronegative for all the markers in the RA panel and served as the controls for the RA+ subjects
- Celiac disease panel-positive (CD+) subjects—these subjects were seropositive for at least one antibody in the celiac disease panel while they might be seropositive for antibodies in the Wheat Zoomer panel as well
- Wheat Zoomer-positive (WZ+) subjects—these subjects were seropositive for at least one antibody in the Wheat Zoomer panel while they were seronegative for any antibody in the celiac disease panel
- Nonwheat-sensitive (NWS) controls—the subjects who were seronegative in both the celiac disease panel and Wheat Zoomer panel and served as the controls for the CD+ or WZ+ subjects

### 2.2. Celiac Disease Panel

Anti-tTG IgG and IgA and anti-DGP IgG and IgA were included in the celiac disease panel (Vibrant America, LLC., San Carlos, CA, USA). This peptide microarray-based assay tested serum specimens as an aid in diagnosis of celiac disease. The fabrication and validation of the peptide arrays are very similar to the manufacture procedures described in our previous work [9, 10]. In general, the immunoassay binding activities between the antibodies in the serum and the attached peptides were scanned by a fluorescence microarray scanner and the data were analyzed to differentiate the substantial levels of binding, which referred to the mean signal binding intensity of the subsequences. The obtained fluorescent binding intensities were then converted to antibody-binding units after normalizing the values for each peptide and compared with an experimentally determined borderline range (0.95-1.05). Any positivity in the celiac disease panel indicates a seropositive celiac disease, except when total IgA was low, it indicates an IgA-deficiency celiac disease.

### 2.3. Wheat Zoomer

Wheat Zoomer (Vibrant America, LLC, San Carlos, CA, USA) testing was performed at Vibrant America, a Clinical Laboratory Improvement Amendments- (CLIA-) and College of American Pathologists- (CAP-) certified laboratory, using ISO-13485-developed technology. Wheat Zoomer is a peptide microarray-based assay covering IgG and IgA antibodies to 14 wheat proteins listed in Table 1. The Wheat Zoomer uses a microchip array containing a wide range of wheat-derived peptides, offering specific recognitions to IgG and IgA. All the key proteins of wheat are arrayed on the Vibrant Wheat Zoomer chip as overlapping 18-mer peptides covering the entire protein. These chips are then placed on a 96-pillar plate and assayed against serum specimens to determine reactivity. The detailed fabrication information for the Wheat Zoomer is very similar to the one as reported in our previous work [9, 10].

### 2.4. Rheumatoid Arthritis (RA) Panel

A RA Panel was performed at Vibrant America Clinical Laboratory (Vibrant America, LLC, San Carlos, CA, USA) and covered anti-RF IgM (Roche Diagnostics, Risch-Rotkreuz, Switzerland) and anti-CCP3 IgG and IgA (Inova Diagnostics, San Diego, CA, USA). The interpretation of the results strictly followed the protocol suggested by the assay provider companies. A serum sample was considered RA negative if the concentrations of the antibodies to all the markers in the panel were equal to or less than the cutoff values. A sample was considered RA positive when it has at least one antibody at borderline of or more than an index value of 0.95.

## 3. Results

### 3.1. Patient Clinical Characteristics

After exclusion of the incomplete clinical data in which some test results were lacking, 844 subjects who ordered the rheumatoid arthritis panel, the celiac disease panel, and the Wheat Zoomer panel were included in this retrospective study.  Table 2 shows the demographics of the positive subjects and negative controls in this study.  Table 3 shows the subjects who were simultaneously seropositive in either RA and CD panels or RA and WZ panels.

### 3.2. Prevalence of RA Markers in CD and WZ Seropositive Subjects and Seronegative Controls

**Figure 1:**
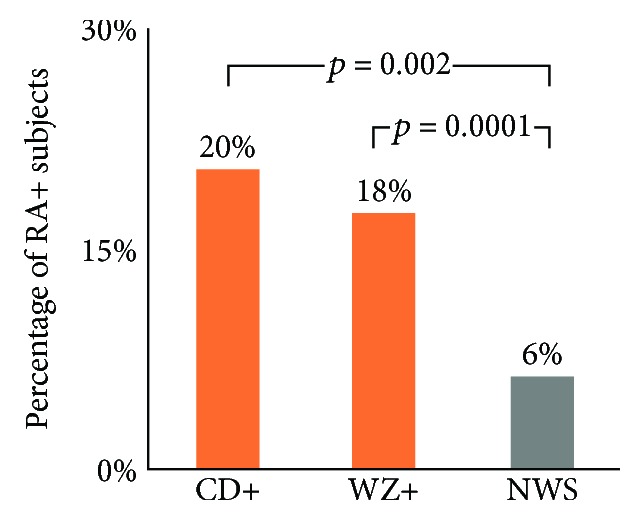
Frequency of RA markers autoantibodies in CD and WZ seropositive subjects.

The prevalence of RA markers, anti-RF IgM and anti-CCP3 IgG and IgA, were investigated in the CD+ subjects, WZ+ subjects, and the NWS seronegative controls, as shown in Figure 1. Among the 49 CD+ subjects, RA markers were found in 10 (20%) subjects. Of the 10 subjects who carry both CD and RA markers, 5 (10%) subjects had anti-CCP3 IgG/IgA and 5 (10%) subjects had anti-RF IgM. Interestingly, the presences of the anti-CCP3 IgG/IgA and anti-RF IgM were complementary and there were no subjects carrying both at the same time. Among 605 WZ+ subjects, RA markers were detected in 106 (18%) subjects. Among these 106 subjects, 76 (12.5%) were seropositive to anti-CCP3 antibody and 37 (6.1%) were seropositive to anti-RF IgM, meaning 7 of the WZ+ subjects carried anti-CCP3 IgG/IgA and anti-RF IgM simultaneously. The control group consisted of 190 NWS subjects who were seronegative in both CD and WZ panels. 12 (6.3%) of the subjects were seropositive in the RA panel. Among them, 8 (4.2%) were positive to anti-CCP while 5 (2.6%) were positive to anti-RF IgM, meaning only one NWS subject had both positive anti-CCP3 IgG/IgA and anti-RF IgM.

### 3.3. Prevalence of CD and WZ Markers in RA Seropositive Subjects and Seronegative Symptomatic Controls

**Figure 2:**
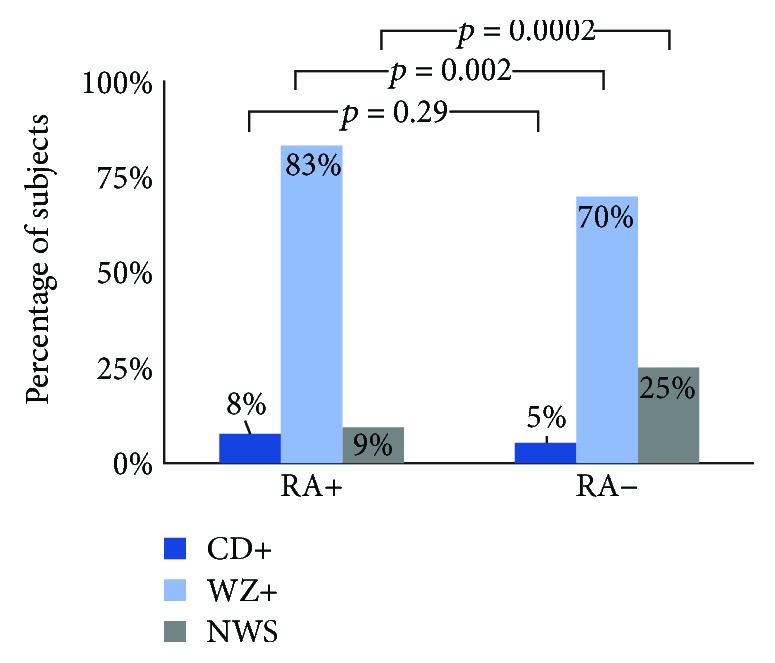
Prevalence of CD and WZ markers in RA-positive and negative-subjects.

The prevalence of CD and WZ markers were intensively researched in the RA seropositive subjects and seronegative symptomatic controls, as shown in Figure 2. In 128 RA+ subjects, 10 (7.8%) were found to have one or more CD hallmark antibodies (anti-tTG IgG, anti-tTG IgA, anti-DGP IgG, anti-DGP IgA). Anti-tTG2 IgG (9/128, 7%) antibodies were the most frequently found CD marker, followed by anti-tTG2 IgA (2/128, 1.6%) and anti-DGP IgA (1/128, 0.8%). All RA+ subjects in this cohort showed negative to anti-DGP IgG.

The frequencies of Wheat Zoomer markers, which are clinically relevant antibodies to significant wheat-related disorders, were also investigated within the same 128 RA+ subjects. 106 (83%) were found to be seropositive for at least one marker in the Wheat Zoomer panel while being seronegative in the CD panel. Moreover, 12 (9%) NWS subjects were found in the RA+ subjects. The prevalence of the same sets of subjects were also investigated in the 716 RA− controls. 39 (5%) CD+ subjects, 499 (70%) WZ+ subjects, and 178 (25%) NWS subjects were found in these symptomatic seronegative controls.

### 3.4. Frequencies of WZ Markers in the RA+ Subjects

**Figure 3:**
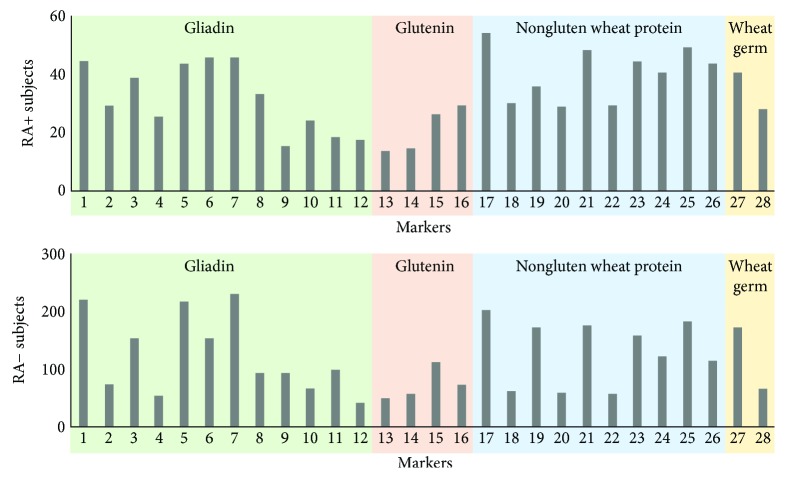
Frequencies of Wheat Zoomer makers in RA+ or RA− subjects.

As shown in Figure 3, among the 128 RA+ subjects, anti-gliadin (95, 74%) antibodies were the most frequently detected WZ antibodies, followed by anti-nongluten wheat protein antibodies (57, 44%), anti-wheat germ (57, 44%), and anti-glutenin (47, 37%). In 716 RA− controls, anti-gliadin (467, 65%) were also the most frequently detected WZ antibodies, followed by anti-nongluten wheat protein antibodies (332, 46%), anti-wheat germ (332, 46%), and anti-glutenin (234, 33%). Figure 3 displays the distribution of each WZ marker in RA+ subjects and RA− controls. See Table S1 in the Supplemental Material section for the antibody labeled by each marker.

## 4. Discussion

The presence of autoantibodies usually indicates significant features in many autoimmune diseases because it may suggest a breakdown of immune tolerance to self-antigens. RA and wheat-related disorders are associated with multiple autoantibodies, respectively, although many of the autoantibodies have been considered to be nonspecific. Understanding the autoantibody production in each population can contribute to answering crucial questions for diagnosing and treating autoimmune diseases. The co-occurrence of celiac-specific and RA-specific autoantibodies have been previously described. Anti-RF and anti-CCP are the two most important autoantibodies in RA that provide different clinical and pathophysiological information. For CD, serum antibodies such as anti-tTG and anti-DGP have been well validated for diagnosis with high sensitivity and specificity. Other than CD, wheat-related disorders can cover a relatively broad spectrum of wheat sensitivity problems, but a definite diagnosis can be problematic except going through restricted food challenge test. A lab-developed test “Wheat Zoomer,” which is a wheat peptide microarray, could detect IgG and IgA reactions to 14 clinically relevant protein markers. Even though the specificity and sensitivity of these markers are still under investigation, we as a clinical laboratory have observed an interesting trend of high prevalence of these markers in the RA patients and reported in here.

We first researched the prevalence of RA markers in subjects who were seropositive in CD and WZ. RA markers were found in 20% CD+ subjects, 18% WZ+ subjects, and 6% NWS controls. There are significantly higher proportions of RA suspects in the seropositive wheat-related subjects than the seronegative controls (*p* < 0.05). Our result is consistent with the numbers reported in a study by Lubrano et al., which consisted of 200 celiac patients and 40 irritable bowel syndrome (IBS) disease controls. Arthritis was found to be present in 26% of the celiac disease group and 7.5% in the IBS control group [5]. However, this result differs from another report, in which the RA's rate was found to be in only 1.8% of the celiac population [15]. Anti-CCP and anti-RF are well-established tests that provide diagnostic and prognostic information for RA patients. In the present study, seropositivity for anti-CCP was found to be slightly higher than anti-RF in the cohort (10% vs 10% in CD+, 12% vs 6.1% in WZ+, and 4.2% vs 2.6% in NWS). These results are in line with several published reports where anti-CCP tests have been shown to be both more sensitive and more specific test for RA than anti-RF [16–19].

The presence of celiac markers in seropositive RA subjects was observed to be 8%, which is close to the 5% observed in the seronegative controls (*p* > 0.05). However, it is noteworthy that 83% of the seropositive RA subjects carry at least one WZ marker, which is significantly greater than the 70% in the seronegative symptomatic controls (*p* < 0.05). The WZ markers are representative immunogenetic wheat proteins excluding the ones specific for CD. A breakdown analysis was applied to IgG and IgA to the 14 species as shown in Figure 3. In this particular cohort, the markers in the gliadin subpanel were significantly more frequent in the RA+ subjects than in RA− symptomatic controls (74% vs 65%, *p* < 0.05) while the markers in the other three subpanels were less significant. The frequency of anti-gliadin antibodies reported in the present study is indeed higher as compared to that in some other studies. Paimela et al. described an increased anti-gliadin antibody level in 37% of RA patients and 12% in spondyloarthropathies controls [20]. In another study, only 9 out of 100 RA (9%) patients were reported to have detectable levels of anti-gliadin antibodies [21]. In another published study consisting of 121 RA patients and 30 primary Sjögren's syndrome controls, anti-gliadin antibodies were detected in only five (4.1%) of the RA patients [22]. We hypothesize that the high frequency of gliadin antibodies in our study is contributed not only to choosing symptomatic cohort but also the utilization of a more sensitive and comprehensive testing tool. While most commercial tests only measure the gliadin and its deamidated forms, the WZ assay covers all known forms of gliadins (i.e., alpha, gamma, and omega gliadins from different wheat species in both native and deamidated forms).

We also scrutinized test results of four patients who tested twice at Vibrant America Clinical Laboratory for the same four tests. The results of the CD and WZ panels were consistent in both visits for all four patients; however, they have seroconverted for the RA panel. All four patients were RA positive in their first visit but converted to negativity at their second visit. We analyzed their wheat protein antibodies and found similar pattern as in the RA panel. These patients were all positive for anti-purinin in their first visit, and three of them converted to negative in the second visit. Purinins are proteins which belong to the nongluten wheat protein family. They have been identified as targets of IgG and/or IgA antibody reactivity in patients with celiac disease [14]. Although the numbers are extremely small to draw any direct conclusions, these findings should prompt further investigation into the potential utility of anti-purinin in the prognosis of RA.

In conclusion, our data presents a strong association of the characteristic serological biomarkers between RA and wheat-related disorders. From the point of view of a clinical laboratory, we observed a significantly greater frequency of RA markers in seropositive subjects with wheat-related disorders as well as wheat protein antibodies in RA subjects compared with the respective seronegative controls. We believe this result is worthy to be explored in more general populations and to be considered while conducting prognosis for both conditions. Accessing disease biomarkers at an early stage will potentially help in lifestyle choices to ameliorate symptoms. Owing to the development of high-throughput quantitative antibody-based assays, pursuing multiple panel testing at an early stage becomes economically possible and could provide enormous potential in improving diagnosis accuracy and predicting disease development.

## Figures and Tables

**Table 1:** Wheat Zoomer protein probes.

Panel	Proteins
Gliadin [11]	*α* gliadin, *α*-*β* gliadin, *γ*-gliadin, *Ω* gliadin, gluteomorphin, prodynorphin
Glutenin [12]	Low molecular weight glutenin, high molecular weight glutenin
Wheat germ [13]	Wheat germ agglutinin
Nongluten wheat protein [14]	Serpin, farnins, amylase/protease inhibitors, globulins, purinin

**Table 2:** Demographics of the studied subjects.

	RA+	RA− (control)	CD+	WZ+	NWS (control)
Number	128	716	49	605	190
Age (*X* ± SD)	47 ± 16	47 ± 16	44 ± 17	48 ± 16	47 ± 16
Gender	91 F/37 M	503 F/213 M	35 F/14 M	426 F/179 M	132 F/58 M

**Table 3:** Demographics of the subjects who were seropositive in two panels.

	RA+/CD+	RA+/WZ+
Number	10	106
Age (*X* ± SD)	46 ± 17	47 ± 16
Gender	6 F/4 M	77 F/29 M

## Data Availability

The data used to support the findings of this study are included within the article.

## Supplementary Materials

Supplementary Materials Table S1: list of marker numbers and the represented antigens.
