# Supplementary material for: Overlap of Characteristic Serological Antibodies in Rheumatoid Arthritis and Wheat-Related Disorders
Source: Dis Markers. 2019 Jan 10;2019:4089178. doi: 10.1155/2019/4089178 (PMC6348907; doi:10.1155/2019/4089178)
Supplement: Supplementary Materials — Table S1: list of marker numbers and the represented antigens. [file 4089178.f1.docx]

**Table S1. List of marker numbers and the represented antigens.**

| **Marker No.** | **Antigen** | **Panel** |
| --- | --- | --- |
| 1 | Alpha Gliadin IgG | Gliadin |
| 2 | Alpha Gliadin IgA | Gliadin |
| 3 | Alpha-Beta Gliadin IgG | Gliadin |
| 4 | Alpha-Beta Gliadin IgA | Gliadin |
| 5 | Gamma Gliadin IgG | Gliadin |
| 6 | Gamma Gliadin IgA | Gliadin |
| 7 | Omega Gliadin IgG | Gliadin |
| 8 | Omega Gliadin IgA | Gliadin |
| 9 | Gluteomorphin IgG | Gliadin |
| 10 | Gluteomorphin IgA | Gliadin |
| 11 | Prodynorphin IgG | Gliadin |
| 12 | Prodynorphin IgA | Gliadin |
| 13 | HMW Glutenin IgG | Glutenin |
| 14 | HMW Glutenin IgA | Glutenin |
| 15 | LMW Glutenin IgG | Glutenin |
| 16 | LMW Glutenin IgA | Glutenin |
| 17 | Serpin IgG | Non-gluten wheat protein |
| 18 | Serpin IgA | Non-gluten wheat protein |
| 19 | Farinins IgG | Non-gluten wheat protein |
| 20 | Farinins IgA | Non-gluten wheat protein |
| 21 | Amylase/Protease Inhibitors IgG | Non-gluten wheat protein |
| 22 | Amylase/Protease Inhibitors IgA | Non-gluten wheat protein |
| 23 | Globulins IgG | Non-gluten wheat protein |
| 24 | Globulins IgA | Non-gluten wheat protein |
| 25 | Purinin IgG | Non-gluten wheat protein |
| 26 | Purinin IgA | Non-gluten wheat protein |
| 27 | Wheat Germ Agglutinin IgG | Wheat germ |
| 28 | Wheat Germ Agglutinin IgA | Wheat germ |
